# (*Z*)-3-(1-Benzofuran-2-yl)-2-(3,4,5-tri­meth­oxy­phen­yl)acrylonitrile

**DOI:** 10.1107/S1600536812005831

**Published:** 2012-02-17

**Authors:** Narsimha Reddy Penthala, Sean Parkin, Peter A. Crooks

**Affiliations:** a Department of Pharmaceutical Sciences, College of Pharmacy, University of Arkansas for Medical Sciences, Little Rock, AR 72205, USA; b Department of Chemistry, University of Kentucky, Lexington, KY 40506, USA

## Abstract

In the title compound, C_20_H_17_NO_4_, the double bond of the acrylonitrile group separating the 1-benzofuran moiety from the 3,4,5-trimeth­oxy­phenyl ring has *Z* geometry. The 1-benzofuran groups are π–π stacked with inversion-related counterparts such that the furan ring centroid–centroid distance is 3.804 (5) Å. The dihedral angle between the planes of the trimeth­oxy­phenyl ring and the acrylonitrile group is 24.2 (2)°.

## Related literature

For the biological activity, see: Naruto *et al.* (1983 ▶); Parmar *et al.* (1988 ▶); Shiba (1996 ▶); Sanna *et al.* (1999 ▶, 2000 ▶); Ohsumi *et al.* (1998 ▶); Saczewski *et al.* (2004 ▶). For similar structures, see: Choi *et al.* (2007 ▶); Seo *et al.* (2009 ▶); Sonar *et al.* (2007 ▶).
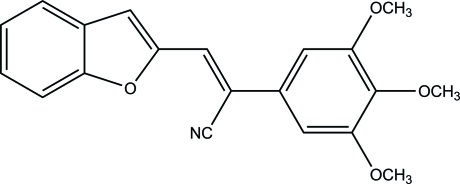


## Experimental

### 

#### Crystal data

C_20_H_17_NO_4_
*M* *_r_* = 335.35
Monoclinic, 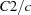

*a* = 28.0892 (5) Å
*b* = 6.9555 (1) Å
*c* = 20.0908 (4) Å
β = 122.678 (1)°
*V* = 3303.93 (10) Å^3^
*Z* = 8
Mo *K*α radiation
μ = 0.09 mm^−1^
*T* = 90 K
0.24 × 0.20 × 0.14 mm

#### Data collection

Nonius KappaCCD diffractometer
26416 measured reflections
3790 independent reflections
2183 reflections with *I* > 2σ(*I*)
*R*_int_ = 0.085

#### Refinement

*R*[*F* ^2^ > 2σ(*F* ^2^)] = 0.064
*wR*(*F* ^2^) = 0.186
*S* = 1.02
3790 reflections
229 parameters
H-atom parameters constrained
Δρ_max_ = 0.51 e Å^−3^
Δρ_min_ = −0.35 e Å^−3^

### 

Data collection: *COLLECT* (Nonius, 1998 ▶); cell refinement: *SCALEPACK* (Otwinowski & Minor, 1997 ▶); data reduction: *DENZO-SMN* (Otwinowski & Minor, 1997 ▶); program(s) used to solve structure: *SHELXS97* (Sheldrick, 2008 ▶); program(s) used to refine structure: *SHELXL97* (Sheldrick, 2008 ▶); molecular graphics: *XP* in *SHELXTL* (Sheldrick, 2008 ▶); software used to prepare material for publication: *SHELXL97* and local procedures.

## Supplementary Material

Crystal structure: contains datablock(s) global, I. DOI: 10.1107/S1600536812005831/hg5136sup1.cif

Structure factors: contains datablock(s) I. DOI: 10.1107/S1600536812005831/hg5136Isup2.hkl

Supplementary material file. DOI: 10.1107/S1600536812005831/hg5136Isup3.cml

Additional supplementary materials:  crystallographic information; 3D view; checkCIF report

## Figures and Tables

**Fig. 1.**
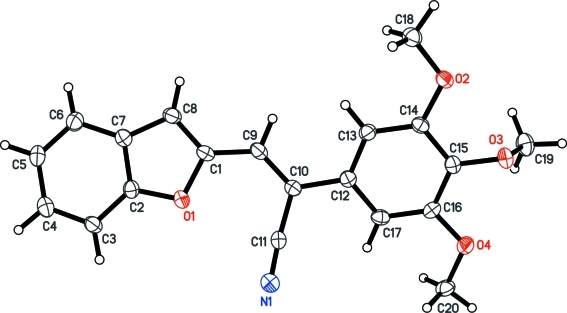
A view of the molecular structure with displacement ellipsoids drawn at the 50% probability level and H atoms shown as small spheres of arbitrary radius.

## supplementary crystallographic information

### Comment

Acrylonitrile analogs that incorporate 1,2,4-triazole, benzimidazole, or 1,3,5-triazine heterocyclic groups have been found to possess interesting biological properties such as spasmolytic (Naruto *et al.*, 1983), antioxidative (Parmar *et al.*, 1988), insecticidal (Shiba, 1996), antitubercular (Sanna *et al.*, 1999, 2000) and cytotoxic (Ohsumi *et al.*, 1998; Saczewski *et al.*, 2004) activities. From our previous studies, we reported the X-ray crystallographic data of two benzothiophene acrylonitrile analogs (Sonar *et al.*, 2007). Based on this, and to compare the structure–activity relationships of different substituted acrylonitrile analogs, we have now prepared the title compound, (I), by the reaction of benzofuran-2-carbaldehyde with 2-(3,4,5-trimethoxyphenyl)acetonitrile in methanolic and sodium methoxide under reflux. The title compound was crystallized from the methanol. The molecular structure is shown in Fig.1. The 1-benzofuran ring is planar, with bond distances and angles comparable with those previously reported for other 1-benzofuran derivatives (Choi *et al.*, 2007; Seo *et al.*, 2009). The X-ray crystallographic studies revealed that the title compound is the *Z* isomer, since the 1-benzofuran ring is *trans* relative to the bulky 3,4,5-trimethoxy phenyl group. The 1-benzofuran groups are π—π stacked with inversion-related (1 - *x*, 1 - *y*, 1 - *z*) counterparts with a furan ring centroid—centroid distance of 3.804 (5) Å. Since the stacked benzofurans are inversion related, they are exactly parallel with perpendicular spacing of 3.409 (3) Å. The dihedral angle between the planes of the trimethoxy phenyl ring and the acrylonitrile group is 24.2 (2) Å.

### Experimental

A mixture of benzofuran-2-carbaldehyde (0.3 g, 2.05 mmol), and 2-(3,4,5-trimethoxyphenyl)acetonitrile (0.45 g, 2.17 mmol) was refluxed in 5% methanolic sodium methoxide solution for 4 hrs. The reaction mixture was cooled to room temperature and added to ice cold water to afford a yellow crude solid, which was collected by filtration, washed with a 1:1 mixture of cold water and methanol, and suction–dried to afford the desired product. Crystallization from methanol gave a yellow crystalline product of (*Z*)-3-(benzofuran-2-yl)-2-(3,4,5-trimethoxyphenyl)acrylonitrile that was suitable for X-ray crystallographic analysis. ^1^H NMR (CDCl_3_): δ 3.90 (s, 3H), 3.91 (s, 6H), 6.89 (s, 2H), 7.26–7.31 (dd, 1H), 7.36–7.40 (m, 1H), 7.41 (s, 1H), 7.50 (s, 1H),7.53–7.56(dd, 1H), 7.63–7. 65 (m, 1H); ^13^C NMR (CDCl_3_): δ 56.53, 61.25, 103.30, 110.89, 111.10, 111.71, 117.60, 122.16, 123.81, 126.94, 127.66, 128.29, 129.17, 139.50, 151.21, 153.68, 155.20.

### Refinement

H atoms were found in difference Fourier maps and subsequently placed in idealized positions with constrained distances of 0.98 Å (RCH_3_), 0.95 Å (C_sp2_H) and with *U*_iso_(H) values set to either 1.2*U*_eq_ or 1.5*U*_eq_ (RCH_3_) of the attached atom.

### Crystal data

**Table:**

C_20_H_17_NO_4_	*F*(000) = 1408
*M**_r_* = 335.35	*D*_x_ = 1.348 Mg m^−^^3^
Monoclinic, *C*2/*c*	Mo *K*α radiation, λ = 0.71073 Å
Hall symbol: -C 2yc	Cell parameters from 4101 reflections
*a* = 28.0892 (5) Å	θ = 1.0–27.5°
*b* = 6.9555 (1) Å	µ = 0.09 mm^−^^1^
*c* = 20.0908 (4) Å	*T* = 90 K
β = 122.678 (1)°	Block, yellow
*V* = 3303.93 (10) Å^3^	0.24 × 0.20 × 0.14 mm
*Z* = 8	

### Data collection

**Table:**

Nonius KappaCCD diffractometer	2183 reflections with *I* > 2σ(*I*)
Radiation source: fine-focus sealed tube	*R*_int_ = 0.085
Graphite monochromator	θ_max_ = 27.5°, θ_min_ = 1.7°
Detector resolution: 9.1 pixels mm^-1^	*h* = −35→36
ω scans at fixed χ = 55°	*k* = −8→9
26416 measured reflections	*l* = −26→25
3790 independent reflections	

### Refinement

**Table:**

Refinement on *F*^2^	Primary atom site location: structure-invariant direct methods
Least-squares matrix: full	Secondary atom site location: difference Fourier map
*R*[*F*^2^ > 2σ(*F*^2^)] = 0.064	Hydrogen site location: inferred from neighbouring sites
*wR*(*F*^2^) = 0.186	H-atom parameters constrained
*S* = 1.02	*w* = 1/[σ^2^(*F*_o_^2^) + (0.1061*P*)^2^] where *P* = (*F*_o_^2^ + 2*F*_c_^2^)/3
3790 reflections	(Δ/σ)_max_ < 0.001
229 parameters	Δρ_max_ = 0.51 e Å^−^^3^
0 restraints	Δρ_min_ = −0.35 e Å^−^^3^

### Special details

**Table:**

Geometry. All e.s.d.'s (except the e.s.d. in the dihedral angle between two l.s. planes) are estimated using the full covariance matrix. The cell e.s.d.'s are taken into account individually in the estimation of e.s.d.'s in distances, angles and torsion angles; correlations between e.s.d.'s in cell parameters are only used when they are defined by crystal symmetry. An approximate (isotropic) treatment of cell e.s.d.'s is used for estimating e.s.d.'s involving l.s. planes.
Refinement. Refinement of *F*^2^ against all reflections. The weighted *R*-value *wR* and goodness of fit *S* are based on *F*^2^. Conventional *R*-values *R* are based on *F*, with *F* set to zero for negative *F*^2^. The threshold expression of *F*^2^ > 2σ(*F*^2^) is used only for calculating *R*-factors(gt) *etc*. and is not relevant to the choice of reflections for refinement. *R*-values based on *F*^2^ are statistically about twice as large as those based on *F*, and *R*-values based on ALL data will be even larger.

### Fractional atomic coordinates and isotropic or equivalent isotropic displacement parameters (Å^2^)

**Table:**

	*x*	*y*	*z*	*U*_iso_*/*U*_eq_	
N1	0.48054 (9)	0.1350 (3)	0.63530 (12)	0.0314 (5)	
O1	0.54675 (6)	0.4633 (2)	0.60175 (9)	0.0235 (4)	
O2	0.28706 (6)	0.8492 (2)	0.58677 (9)	0.0236 (4)	
O3	0.26167 (6)	0.5664 (2)	0.65217 (9)	0.0241 (4)	
O4	0.33056 (7)	0.2610 (2)	0.71961 (10)	0.0267 (4)	
C1	0.52309 (9)	0.6378 (3)	0.60312 (13)	0.0209 (5)	
C2	0.58856 (9)	0.5094 (3)	0.58841 (12)	0.0205 (5)	
C3	0.62441 (10)	0.3797 (3)	0.58480 (13)	0.0246 (6)	
H3	0.6209	0.2451	0.5890	0.030*	
C4	0.66571 (10)	0.4553 (3)	0.57475 (13)	0.0251 (6)	
H4	0.6916	0.3717	0.5727	0.030*	
C5	0.66959 (10)	0.6539 (4)	0.56754 (13)	0.0261 (6)	
H5	0.6984	0.7025	0.5610	0.031*	
C6	0.63306 (10)	0.7803 (4)	0.56964 (14)	0.0275 (6)	
H6	0.6360	0.9145	0.5637	0.033*	
C7	0.59119 (9)	0.7082 (3)	0.58073 (13)	0.0223 (5)	
C8	0.54814 (10)	0.7870 (3)	0.59013 (13)	0.0238 (6)	
H8	0.5388	0.9192	0.5877	0.029*	
C9	0.47910 (9)	0.6394 (3)	0.61858 (13)	0.0224 (5)	
H9	0.4655	0.7636	0.6198	0.027*	
C10	0.45380 (9)	0.4932 (3)	0.63175 (12)	0.0195 (5)	
C11	0.47002 (10)	0.2961 (3)	0.63316 (13)	0.0227 (5)	
C12	0.40551 (9)	0.5193 (3)	0.64184 (12)	0.0192 (5)	
C13	0.37106 (9)	0.6807 (3)	0.61109 (13)	0.0212 (5)	
H13	0.3796	0.7790	0.5863	0.025*	
C14	0.32409 (9)	0.6977 (3)	0.61670 (12)	0.0194 (5)	
C15	0.31095 (9)	0.5540 (3)	0.65306 (12)	0.0203 (5)	
C16	0.34635 (9)	0.3953 (3)	0.68529 (12)	0.0208 (5)	
C17	0.39355 (9)	0.3767 (3)	0.68009 (13)	0.0217 (5)	
H17	0.4176	0.2678	0.7024	0.026*	
C18	0.29675 (10)	0.9924 (3)	0.54421 (13)	0.0260 (6)	
H18A	0.3342	1.0497	0.5790	0.039*	
H18B	0.2678	1.0926	0.5258	0.039*	
H18C	0.2949	0.9330	0.4986	0.039*	
C19	0.26949 (10)	0.6446 (4)	0.72368 (14)	0.0314 (6)	
H19A	0.2982	0.5697	0.7691	0.047*	
H19B	0.2337	0.6388	0.7211	0.047*	
H19C	0.2819	0.7787	0.7296	0.047*	
C20	0.36866 (10)	0.1064 (3)	0.75978 (14)	0.0260 (6)	
H20A	0.3713	0.0270	0.7216	0.039*	
H20B	0.3549	0.0278	0.7865	0.039*	
H20C	0.4061	0.1578	0.7990	0.039*	

### Atomic displacement parameters (Å^2^)

**Table:**

	*U*^11^	*U*^22^	*U*^33^	*U*^12^	*U*^13^	*U*^23^
N1	0.0334 (13)	0.0259 (13)	0.0429 (13)	0.0016 (10)	0.0257 (11)	−0.0004 (10)
O1	0.0229 (9)	0.0234 (9)	0.0289 (9)	0.0020 (7)	0.0170 (8)	−0.0005 (7)
O2	0.0240 (9)	0.0228 (9)	0.0273 (9)	0.0060 (7)	0.0161 (8)	0.0043 (7)
O3	0.0194 (9)	0.0304 (10)	0.0251 (9)	0.0007 (7)	0.0136 (7)	−0.0019 (7)
O4	0.0247 (9)	0.0252 (10)	0.0334 (9)	0.0018 (7)	0.0178 (8)	0.0077 (7)
C1	0.0202 (12)	0.0174 (12)	0.0225 (12)	0.0031 (10)	0.0100 (10)	−0.0011 (10)
C2	0.0174 (12)	0.0239 (13)	0.0198 (12)	−0.0016 (10)	0.0097 (10)	−0.0023 (10)
C3	0.0281 (14)	0.0211 (13)	0.0274 (13)	0.0042 (11)	0.0168 (12)	−0.0002 (10)
C4	0.0224 (13)	0.0326 (15)	0.0208 (12)	0.0045 (11)	0.0121 (11)	0.0009 (11)
C5	0.0229 (13)	0.0344 (15)	0.0248 (13)	0.0004 (11)	0.0155 (11)	0.0016 (11)
C6	0.0293 (14)	0.0260 (14)	0.0326 (14)	−0.0035 (11)	0.0203 (12)	−0.0019 (11)
C7	0.0207 (12)	0.0231 (13)	0.0229 (12)	0.0009 (10)	0.0118 (10)	−0.0008 (10)
C8	0.0234 (13)	0.0187 (13)	0.0285 (13)	0.0028 (10)	0.0136 (11)	0.0001 (10)
C9	0.0206 (12)	0.0205 (13)	0.0258 (13)	0.0027 (10)	0.0123 (11)	−0.0033 (10)
C10	0.0204 (12)	0.0186 (12)	0.0193 (11)	0.0021 (10)	0.0107 (10)	−0.0001 (10)
C11	0.0218 (13)	0.0237 (14)	0.0273 (13)	0.0000 (11)	0.0163 (11)	−0.0001 (11)
C12	0.0180 (12)	0.0195 (12)	0.0184 (11)	−0.0021 (10)	0.0086 (10)	−0.0035 (10)
C13	0.0247 (13)	0.0192 (13)	0.0224 (12)	−0.0006 (10)	0.0144 (11)	0.0004 (10)
C14	0.0200 (12)	0.0184 (12)	0.0181 (11)	0.0013 (10)	0.0092 (10)	−0.0016 (9)
C15	0.0183 (12)	0.0229 (13)	0.0193 (12)	−0.0008 (10)	0.0100 (10)	−0.0024 (10)
C16	0.0229 (13)	0.0195 (13)	0.0193 (12)	−0.0038 (10)	0.0109 (11)	0.0001 (10)
C17	0.0215 (12)	0.0190 (13)	0.0204 (12)	0.0029 (10)	0.0086 (10)	0.0027 (10)
C18	0.0267 (13)	0.0260 (14)	0.0256 (13)	0.0037 (11)	0.0142 (11)	0.0023 (11)
C19	0.0293 (14)	0.0412 (16)	0.0279 (14)	0.0019 (12)	0.0181 (12)	−0.0012 (12)
C20	0.0320 (14)	0.0211 (13)	0.0271 (13)	0.0000 (11)	0.0173 (12)	0.0045 (10)

### Geometric parameters (Å, º)

**Table:**

N1—C11	1.154 (3)	C8—H8	0.9500
O1—C2	1.376 (3)	C9—C10	1.346 (3)
O1—C1	1.391 (3)	C9—H9	0.9500
O2—C14	1.371 (3)	C10—C11	1.440 (3)
O2—C18	1.431 (3)	C10—C12	1.487 (3)
O3—C15	1.377 (3)	C12—C13	1.390 (3)
O3—C19	1.438 (3)	C12—C17	1.402 (3)
O4—C16	1.370 (3)	C13—C14	1.388 (3)
O4—C20	1.421 (3)	C13—H13	0.9500
C1—C8	1.356 (3)	C14—C15	1.400 (3)
C1—C9	1.428 (3)	C15—C16	1.390 (3)
C2—C3	1.383 (3)	C16—C17	1.391 (3)
C2—C7	1.397 (3)	C17—H17	0.9500
C3—C4	1.384 (3)	C18—H18A	0.9800
C3—H3	0.9500	C18—H18B	0.9800
C4—C5	1.400 (3)	C18—H18C	0.9800
C4—H4	0.9500	C19—H19A	0.9800
C5—C6	1.369 (3)	C19—H19B	0.9800
C5—H5	0.9500	C19—H19C	0.9800
C6—C7	1.403 (3)	C20—H20A	0.9800
C6—H6	0.9500	C20—H20B	0.9800
C7—C8	1.430 (3)	C20—H20C	0.9800
			
C2—O1—C1	105.54 (17)	C13—C12—C10	120.4 (2)
C14—O2—C18	116.95 (17)	C17—C12—C10	119.6 (2)
C15—O3—C19	113.60 (17)	C14—C13—C12	119.8 (2)
C16—O4—C20	116.92 (17)	C14—C13—H13	120.1
C8—C1—O1	111.17 (19)	C12—C13—H13	120.1
C8—C1—C9	129.5 (2)	O2—C14—C13	124.0 (2)
O1—C1—C9	119.36 (19)	O2—C14—C15	115.23 (19)
O1—C2—C3	125.5 (2)	C13—C14—C15	120.8 (2)
O1—C2—C7	110.68 (19)	O3—C15—C16	121.12 (19)
C3—C2—C7	123.8 (2)	O3—C15—C14	119.68 (19)
C2—C3—C4	116.8 (2)	C16—C15—C14	119.1 (2)
C2—C3—H3	121.6	O4—C16—C15	115.57 (19)
C4—C3—H3	121.6	O4—C16—C17	123.7 (2)
C3—C4—C5	120.6 (2)	C15—C16—C17	120.7 (2)
C3—C4—H4	119.7	C16—C17—C12	119.7 (2)
C5—C4—H4	119.7	C16—C17—H17	120.2
C6—C5—C4	122.0 (2)	C12—C17—H17	120.2
C6—C5—H5	119.0	O2—C18—H18A	109.5
C4—C5—H5	119.0	O2—C18—H18B	109.5
C5—C6—C7	118.8 (2)	H18A—C18—H18B	109.5
C5—C6—H6	120.6	O2—C18—H18C	109.5
C7—C6—H6	120.6	H18A—C18—H18C	109.5
C2—C7—C6	118.1 (2)	H18B—C18—H18C	109.5
C2—C7—C8	105.4 (2)	O3—C19—H19A	109.5
C6—C7—C8	136.5 (2)	O3—C19—H19B	109.5
C1—C8—C7	107.2 (2)	H19A—C19—H19B	109.5
C1—C8—H8	126.4	O3—C19—H19C	109.5
C7—C8—H8	126.4	H19A—C19—H19C	109.5
C10—C9—C1	130.3 (2)	H19B—C19—H19C	109.5
C10—C9—H9	114.8	O4—C20—H20A	109.5
C1—C9—H9	114.8	O4—C20—H20B	109.5
C9—C10—C11	121.9 (2)	H20A—C20—H20B	109.5
C9—C10—C12	123.5 (2)	O4—C20—H20C	109.5
C11—C10—C12	114.56 (19)	H20A—C20—H20C	109.5
N1—C11—C10	175.8 (2)	H20B—C20—H20C	109.5
C13—C12—C17	120.0 (2)		
			
C2—O1—C1—C8	0.8 (2)	C9—C10—C12—C17	−159.1 (2)
C2—O1—C1—C9	−177.95 (18)	C11—C10—C12—C17	23.9 (3)
C1—O1—C2—C3	178.0 (2)	C17—C12—C13—C14	−1.6 (3)
C1—O1—C2—C7	−0.5 (2)	C10—C12—C13—C14	176.09 (19)
O1—C2—C3—C4	−176.8 (2)	C18—O2—C14—C13	3.0 (3)
C7—C2—C3—C4	1.5 (3)	C18—O2—C14—C15	−175.70 (18)
C2—C3—C4—C5	−0.9 (3)	C12—C13—C14—O2	−178.6 (2)
C3—C4—C5—C6	−0.4 (4)	C12—C13—C14—C15	0.0 (3)
C4—C5—C6—C7	1.1 (4)	C19—O3—C15—C16	85.9 (2)
O1—C2—C7—C6	177.78 (19)	C19—O3—C15—C14	−98.1 (2)
C3—C2—C7—C6	−0.8 (3)	O2—C14—C15—O3	4.1 (3)
O1—C2—C7—C8	0.0 (2)	C13—C14—C15—O3	−174.67 (19)
C3—C2—C7—C8	−178.5 (2)	O2—C14—C15—C16	−179.78 (19)
C5—C6—C7—C2	−0.6 (3)	C13—C14—C15—C16	1.5 (3)
C5—C6—C7—C8	176.3 (3)	C20—O4—C16—C15	−174.09 (19)
O1—C1—C8—C7	−0.8 (3)	C20—O4—C16—C17	7.6 (3)
C9—C1—C8—C7	177.8 (2)	O3—C15—C16—O4	−3.8 (3)
C2—C7—C8—C1	0.5 (3)	C14—C15—C16—O4	−179.84 (19)
C6—C7—C8—C1	−176.6 (3)	O3—C15—C16—C17	174.64 (19)
C8—C1—C9—C10	−179.9 (2)	C14—C15—C16—C17	−1.4 (3)
O1—C1—C9—C10	−1.3 (4)	O4—C16—C17—C12	178.18 (19)
C1—C9—C10—C11	0.9 (4)	C15—C16—C17—C12	−0.1 (3)
C1—C9—C10—C12	−175.8 (2)	C13—C12—C17—C16	1.6 (3)
C9—C10—C12—C13	23.2 (3)	C10—C12—C17—C16	−176.07 (19)
C11—C10—C12—C13	−153.8 (2)		
